# Propolis and its therapeutic effects on renal diseases: A review

**DOI:** 10.22038/IJBMS.2024.73081.15880

**Published:** 2024

**Authors:** Fatemeh Salami, Reza Mohebbati, Sara Hosseinian, Samira Shahraki, Hossein Hossienzadeh, Abolfazl Khajavi Rad

**Affiliations:** 1 Department of Physiology, Faculty of Medicine, Mashhad University of Medical Sciences, Mashhad, Iran; 2 Department of Physiology, Faculty of Medicine, Gonabad University of Medical Sciences, Gonabad, Iran; 3 Applied Biomedical Research Center, Mashhad University of Medical Sciences, Mashhad, Iran; 4 Department of Physiology, Faculty of Medicine, Zahedan University of Medical Sciences, Zahedan, Iran; 5 Department of Pharmacodynamics and Toxicology, School of Pharmacy, Mashhad University of Medical Sciences, Mashhad, Iran; 6 Pharmaceutical Research Center, Pharmaceutical Technology Institute, Mashhad University of Medical Sciences, Mashhad, Iran

**Keywords:** Anti-inflammatory, Anti-oxidants, Kidney, Propolis, Toxicity

## Abstract

Propolis is produced by bees using a mixture of bees wax and saliva. It contains several bioactive compounds that mainly induce anti-oxidant and anti-inflammatory effects. In this review, we aimed to investigate the effects of propolis on kidney diseases. We used “Kidney”, “Disease”, “Propolis”, “Renal”, “Constituent”, “Mechanism”, “Infection”, and other related keywords as the main keywords to search for works published before July 2023 in Google scholar, Scopus, and Pubmed databases. The search terms were selected according to Medical Subject Headings (MeSH). This review showed that propolis affects renal disorders with inflammatory and oxidative etiology due to its bioactive compounds, mainly flavonoids and polyphenols. There have been few studies on the effects of propolis on kidney diseases; nevertheless, the available studies are integrated in this review. Overall, propolis appears to be effective against several renal diseases through influencing mechanisms such as apoptosis, oxidative balance, and inflammation.

## Introduction

Chronic kidney disease (CKD) and acute kidney injury (AKI) are associated with high mortality and morbidity. AKI is defined as rapid decline in renal function and is related to accelerated CKD (1). Factors such as drugs (e.g., cisplatin through proximal tubular injury, oxidative stress, and inflammation) (2), toxins (e.g., aristolochic acid through proximal epithelial cytotoxic effects and tubular atrophy) (3), sepsis, and ischemia-reperfusion (IR) result in AKI, decreased glomerular filtration rate (GFR), and tubular cell death (4). CKD is a global medical problem and is diagnosed in the presence of significant albuminuria or an estimated GFR of <60 mL/min/1.73 m^2^ (5). Conditions such as diabetes, hypertension, vascular disease, and glomerulonephritis are considered CKD risk factors (6). TGF-β is a key factor in CKD, exerting several effects on renal compartments. TGF-β is produced by most renal cells when they are activated by auto or paracrine mediators such as hormones, cytokines, and chemokines. TGF-β adversely affects the kidneys in pathologies (7). One of the major signs of CKD is interstitial fibrosis characterized by scar-forming myofibroblasts. Resident fibroblasts and pericytes that differentiate into myofibroblasts are principal contributors to fibrosis (8-10). Various inflammatory cells including monocytes, neutrophils, lymphocytes, and dendritic cells that invade the kidney are involved in the repair process after AKI (11, 12). Anti-inflammatory drugs and inhibitors of ROS (reactive oxygen species) production exert reno-protective effects in experimental models of nephropathy (13, 14). 

Many natural products have been shown to alleviate kidney disease by reducing oxidative stress and inflammation (15). Honey bees provide a variety of organic substances, including beeswax, propolis, and royal jelly, which serve as valuable sources of medicine and nutrition (16). 

Propolis is produced by bees using a mixture of beeswax and saliva. This compound acts as a defense mechanism for the hive (17). Because of its bioactivity and health benefits, propolis has been broadly researched in the scientific literature (18-21). The presence of various bioactive phytochemicals such as phenolic acids, ﬂavonoids, esters, diterpenes, aromatic aldehydes, amino acids, fatty acids, vitamins, and minerals confers anti-oxidant properties to propolis (22). The composition of propolis varies among hives, locations, and seasons. Given that propolis functions as a support for hive health, the defensive bioactive compounds found in propolis could also provide benefits for human health (18, 19). The most frequently investigated health benefits of propolis are its anti-microbial, wound healing, and cardio-protective effects. Propolis includes a variety of phenolic compounds, mainly ﬂavonoids and phenolic acids, which can support human health through their anti-oxidant and inﬂammatory activities (23, 24). The ﬂavonoids, phenolic acids and their esters, terpenoids, steroids, and amino acids present in propolis have exhibited anti-inﬂammatory activity. Several mechanisms are involved in the anti-inflammatory effects of propolis including the inhibition of cyclooxygenase (COX) and prostaglandin biosynthesis, free radical scavenging, inhibition of nitric oxide synthesis, the reduction of inﬂammatory cytokines, and immunosuppressive activity (18). Due to the multitude of bioactive components it contains, propolis finds application in the treatment of various ailments (25). This review aims to explore the effects of propolis on kidney diseases.

## Materials and Methods

This review is conducted using comprehensive data. Google Scholar, Scopus, and Pubmed were searched using “Kidney”, “Disease”, “Propolis”, “Renal”, “Constituent “, “Mechanism”, “Infection”, and other related keywords as the main search terms. All records published in English before July 2023 were included in the review and those published in Persian or with only their abstracts available were excluded. The keywords were selected based on Medical Subject Headings (MeSH); the search was performed using the keywords independently and in combination.

## Results

***Propolis constituents***

The composition of propolis depends on the source plant and collection time (26). The main ingredients are resin (40%–50%), wax (25%–30%), essential oils (8%–10%), bee pollen (3%–5%), organic acids and amino acids (1%–3%), and vitamins and minerals (1%) (27-29). Propolis contains various polyphenols such as flavonoids, flavonols, and phenolic acids. The main active ingredients include caffeic acid phenethyl ester (CAPE), galangin, chrysin, nemorosone, propolin G, artepillin C, cardanol, pinocembrin, pinobanksin, chicoric acid, and phenolic acids (caffeic acid, ferulic acid, and coumaric acid), as well as luteolin, apigenin, myricetin, naringenin, kaempferol, quercetin, polysaccharides, tannins, terpenes, sterols, and aldehydes (27, 30-32) (Figure 2). 

High-performance liquid chromatography (HPLC) shows that the phenolic content of propolis is a combination of chrysin, galangin, pinostrobin, pinobanksin, and pinocembrin (33, 34). The biological effects of propolis include anti-inflammatory, anti-oxidant, anti-cancer, and anti-diabetes due to the presence of various organic acids, vitamins (C, A, and B complex), and minerals (Ca, P, Mg, Fe, and K), as well as active polyphenols, which are used as therapeutic products (35).

***Propolis and diabetic nephropathy***

**Figure 1:**
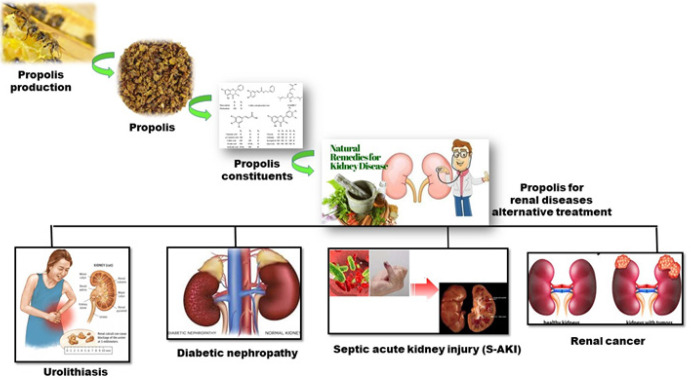
Graphical abstract

In diabetes mellitus, elevated serum glucose and free radicals can induce renal glomerulosclerosis and tubulointerstitial injury leading to diabetic nephropathy (37-39). Propolis treatment causes a significant amelioration in body and kidney weight and leads to a potent free radical scavenging effect (40). In diabetic rats, serum BUN and creatinine are significantly increased. Treatment with propolis significantly decreases BUN; however, creatinine declined only with the middle and high doses of propolis. Furthermore, urinary albumin excretion, a marker for diabetic nephropathy, was ameliorated after treatment with propolis in a dose-dependent manner. Therefore, propolis can attenuate renal injury in diabetic rats (41) (Figure 1). Furthermore, CAPE improved renal function in a rat model with renal tubular damage and oxidative stress induced by lithium (42). In this study, serum levels of glucose, total cholesterol, LDL-C, TG, and MDA were significantly increased in diabetic rats compared to the controls. Propolis treatment at different doses significantly ameliorated these parameters dose-dependently (41). 

According to studies conducted on animals, it has been determined that the appropriate dosage of propolis for individuals in good health is 1.4 mg/kg/day or a total of 70 mg /day (43). The LD50 of propolis extract for mice surpasses 7.34 g/kg, so the safety of therapeutic dosages for humans is ensured. (44).  Mohammadzadeh *et al*. demonstrated that the oral administration of hydroalcoholic propolis extract in rats, at various dosage levels (4.5, 9, 13, and 20 g/kg b.wt), has no toxic effects (45). It is challenging to determine the appropriate dosage of propolis due to the varying levels of product purity and the variations in phenolic compounds found in propolis (23).

 Ethanolic and aqueous propolis extractions can reduce glucose, MDA, nitric oxide, total cholesterol, TG, LDL-C, and VLDL-C and increase HDL- C and SOD in diabetic rats (46). In a study by Jessica *et al*., the effects of pinocembrin were investigated using preventive (i.e., before renal damage) and corrective (i.e., once renal damage is established) schemes in rats with diabetic nephropathy. Treatment with pinocembrin in the preventive scheme ameliorated lipid profile, proteinuria, and glomerular filtration rate and mitigated oxidative stress, thickness of glomerular basement membrane, urinary biomarkers, and delayed death. In the corrective scheme, however, pinocembrin only improved lipid profile while aggravating kidney damage. Based on the above data, the pinocembrin isolated from Mexican brown propolis only improved diabetic nephropathy without kidney damage in a preventive scheme. The underlying mechanism of the protective effect of propolis is the reduction of oxidative stress, which is identified as a major cause of initiation and development of renal damage (47). In an animal study on diabetes-induced nephropathy, Malaysian propolis triggered significant decreases in the activity of renal anti-oxidant enzymes, total anti-oxidant capacity, chloride, and serum sodium levels, and increased serum creatinine, urea, uric acid, and kidney lactate dehydrogenase activity in diabetic rats. Therefore, Malaysian propolis had beneficial effects on renal function in diabetic rats (48).

Researchers in 2007 reported that lipid profile, MDA, and SOD activity were improved by propolis treatment in healthy women and men (49, 50). According to Abo-Salema *et al*., elevated renal MDA content and decreased GSH, SOD, and CAT activity were observed in diabetic rats compared to the control group; however, propolis treatment significantly improved these parameters (41). Although podocyte loss is a characteristic of early-phase diabetic renal disease, it may be imputed to impaired autophagy in diabetes, which eventually leads to proteinuria in diabetic nephropathy. Chrysin, a flavonoid in propolis, inhibits diabetes-associated podocyte injury following exposure to high glucose levels, thus exhibiting an anti-proteinuria effect. The anti-inflammatory effect of chrysin may be responsible for its effectiveness against podocyte injury (51). MCP-1 is a cytokine that improves monocyte recruitment and transformation into macrophages; MCP-1 levels correlate directly with the progression of CKD (52). According to some studies, MCP-1 receptor blockers suppress inflammation while stimulation of MCP-1 synthesis is related to oxidative stress pathways and protein kinase C (52-54). It has been shown that propolis causes a progressive reduction in urinary MCP-1 over 12 months of treatment, which could contribute to proteinuria mitigation (55).

***Propolis and renal cancer***

Turkish propolis has shown anticarcinogenic properties (56, 57) (Figure 1), its antitumor effect being due to the flavonoids that inhibit DNA synthesis (58). Mechanisms such as cell-cycle arrest, induction of apoptosis, and inhibition of cancer cell proliferation are related to the antitumor properties of propolis (59-61). Also, the anticancer activity of propolis is ascribed to its ability to inhibit the localization of NF-κB and regulate gene expression (62). Propolis inhibits cancer development by targeting several signaling pathways such as mitogen-activated protein kinase (MAPK) and phosphoinositide 3-kinases (PI3K)/Akt signaling pathways (62). It has been shown that MeOH propolis extract prevents human renal cell carcinoma (RCC) proliferation *in vitro,* although its molecular mechanisms of action are not fully understood. Phenolic compounds of propolis have anti-oxidant effects which may play a key role in its anticancer properties. Anti-proliferative effects of the caffeic acid derivatives in propolis are probably connected to its modulation of oxidative processes in cells (63, 64). In one study on lipid peroxidation and carcinogenesis induced by Fe-NTA, the oral intake of propolis showed the most potent inhibitory effect. Also, artepillin C had inhibitory effects on the proliferation of cancer cells and induced instant apoptosis in mice tumor cells. Therefore, artepillin C is a potential bioavailable option for chemoprevention of degenerative diseases since it has a strong affinity for attaching to cell membranes, which is difficult to conjugate (65). Moreover, propolis extract has an anti-proliferative effect on human renal cancer cells. Comparison of* in vitro* reactions of normal and cancerous cells to propolis extracts reveals that cancerous cells are more sensitive than normal cells. Portuguese propolis is particularly considered a therapeutic agent in the prevention of diseases caused by free radicals and RCC (66). Fractions of Portuguese propolis (n-hexane, ethyl acetate, n-butanol, and water) show strong toxicity on Caki-2, 786-O, and A498 kidney cell carcinoma cell lines. These results illustrate the potential of propolis and its constituents as promising coadjuvants in the treatment of kidney cancer (67). Wnt signaling is a fundamental and developmentally conserved pathway that regulates cell proliferation, migration, and differentiation. β-catenin, a downstream molecule in the Wnt pathway, and two of its suppressors (APC and axin) are involved in cancer development. Mutation of the β-catenin gene is associated with some cancers (68). Another study shows that caffeic acid inhibits the angiogenesis of human kidney tumors implanted in nude mice. The decrease in VEGF and diminishment of tumor development are attributed to the inhibition of STAT phosphorylation and reduction of HIF-1-mediated expression of VEGF (62, 69).

***Propolis and urolithiasis***

Urolithiasis is a common condition in preliminary health care which can affect all people (70). Propolis has anti-inflammatory, antimicrobial, anti-oxidant, renoprotective, and immune-modulatory effects. There is a close relationship between inflammation and urinary calculous formation (71). Propolis shows anti-inflammatory activity due to the presence of active flavonoids such as CAPE (72, 73). The flavonoids in propolis are potent anti-oxidants (74) and the role of oxidative damage in the pathophysiology of urolithiasis has been shown (75). A study investigating the anti-oxidant, reversing, and protective effects of propolis *in vivo* revealed a dramatic reduction of crystal deposition and efficient inhibition of oxalate-induced renal injury after treatment. Researchers demonstrated the preventive role of anti-oxidants in stone formation (76). In renal ischemia-reperfusion injury, acute administration of CAPE lowers oxidative stress indices (77). Given the natural origin of propolis and its minimal harmful effects, it is valuable as a new treatment for urolithiasis (Figure 1) (78).

***Propolis and septic acute kidney injury (S-AKI)***

Sepsis is a systemic disease caused by runaway reaction to infection (79). Sepsis is an important risk factor for acute renal failure (80). Inflammatory mediators may affect renal tubular cells, causing renal damage. Apoptosis is considered a key factor in septic kidney failure. During sepsis, Bcl-2 up-regulation prevents lymphocyte apoptosis (81). Züleyha et al. studied the protective effect of propolis against kidney damage in a rat model of sepsis induced by LPS. They report that treatment with propolis ameliorated oxidation damage in the kidney via anti-oxidant activity (82). It is reported that red propolis can reduce kidney hypertension, proteinuria, serum creatinine, macrophage infiltration, and renal oxidative stress in a 5/6 renal ablation animal model (83). A study on the effects of green propolis on acute kidney injury in a rat model of sepsis showed that propolis improved survival, reduced sepsis-induced AKI, and restored renal tubular function via down-regulation of the Toll-like receptor 4/nuclear factor-kappa B axis, decreasing inflammatory cytokine levels, and macrophage infiltration in renal tissues. Therefore, treatment with propolis can preserve endothelial function, diminish oxidative stress, and modulate inflammation (84) (Figure 1). Pinocembrin lowers the levels of pro-inflammatory cytokines (e.g., TNF-α), chemokines, and inducible nitric oxide synthase (iNOS) in rats with middle cerebral artery occlusion (MCAO) (20). Pinocembrin could suppress NF-κB and down-regulate TNF-α expression in a mouse model of diabetic encephalopathy (DE) (85).

**Figure 2:**
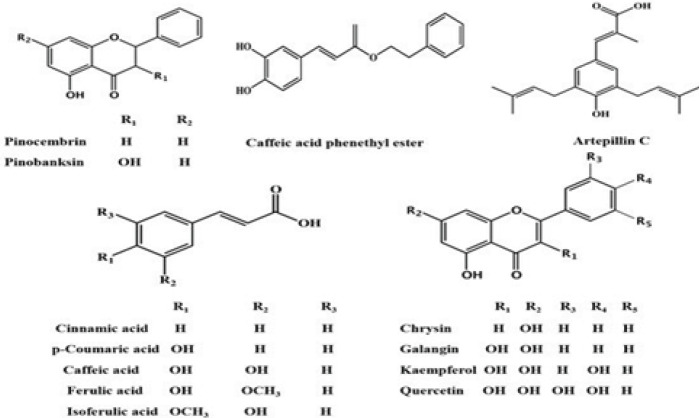
Chemical components of propolis (36)

**Figure 3:**
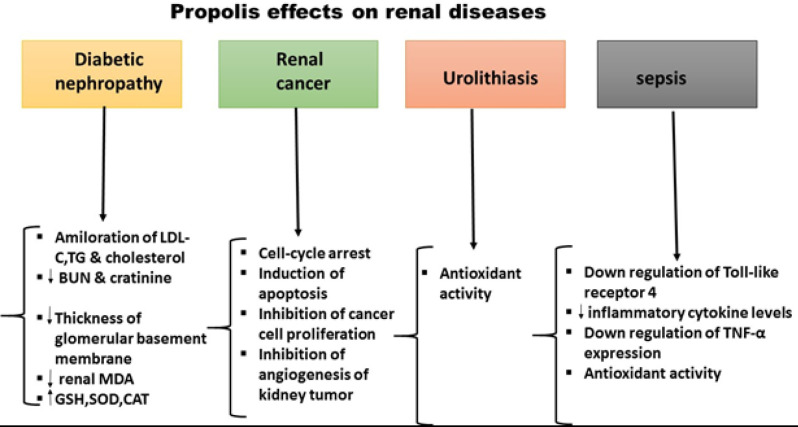
Mechanisms underlying the effects of propolis on some renal diseases

**Table 1:** Effects of propolis on some renal diseases

Disease	Ingredient	Administrated dose	Species	Effects	Reference
Diabetes	Propolis	100, 200و and 300 mg/kg	Rat	-Serum BUN and creatinine were significantly increased -Urinary albumin excretion was ameliorated	(38)
Diabetes	Caffeic acid phenethyl ester	10 µM/kg/day	Rat	-Improved renal function in a rat model with renal tubular damage and oxidative stress induced by lithium	(39)
Diabetes	Ethanolic and aqueous propolis extracts	1 ml/100 g	Rat	-Reduced glucose, MDA, nitric oxide, total cholesterol, TG, and LDL-C VLDL-C -Increased HDL-C and SOD in diabetic rats	(40)
Diabetes	Propolis	Total daily dose of 48.75 mg of flavonoids	Mice Human	-Improved lipid profile, MDA, and SOD activity in healthy women and men	(41,42)
Diabetes	Chrysin	10 mg/kg/day	Mice	-Decreased apoptosis of podocytes exposed to high glucose levels -Anti-proteinuria effect	(43)
Diabetes	Brazilian green propolis extract	500 mg/day	Human	-Progressive reduction in urinary MCP-1 over 12 months of treatment	(47)
Renal cancer	MeOH propolis extract	3.5 µg/ml 8.1 and 6.7 µg/ml	Cell line Cell line	-Prevented the proliferation of human renal cell carcinoma (RCC) *in vitro*	(54, 55)
Septic acute kidney injury (S-AKI)	Red propolis	150 mg/kg/day	Rat	-Reduces kidney hypertension, proteinuria, serum creatinine, macrophage infiltration, and renal oxidative stress in a 5/6 renal ablation animal model	(68)
Septic acute kidney injury (S-AKI)	Pinocembrin	10 mg/kg 50 mg/kg	Rat Mice	-Reduced pro-inflammatory cytokines (TNF-α, interleukin-1beta (IL-1β)), chemokines, inducible nitric oxide synthase (iNOS), and aquaporin-4 in rats with middle cerebral artery occlusion (MCAO) -Suppressed NF-κB and down-regulated TNF-α expression in a diabetic encephalopathy (DE) mouse model	(18)

## Discussion

Oxidative stress is an important pathway in the progression of CKD (86, 87). Anti-oxidant agents have shown reno-protective properties in experimental studies (88). It has been shown that propolis has reno-protective effects in animal models. In a streptozotocin-induced rat model of diabetes, propolis decreased malondialdehyde (MDA) and elevated the activity of anti-oxidants such as glutathione (GSH), superoxide dismutase (SOD), and catalase (CAT) (41, 89). Furthermore, it is reported that propolis has anti-oxidant potential (73, 90). The anti-oxidant properties of propolis are dose-dependent and related to the polyphenol content of its extract (41, 91).

Propolis has anti-inflammatory, anti-oxidant, anti-fibrosis, anti-tumor, antibacterial, anti-fungal, anti-parasitic, and antiviral properties. Because of its extensive therapeutic activities, propolis has been used to treat various diseases for centuries (92). Propolis exerts a protective effect on the integrity of the renal tissue membrane. According to Bhadauria (2012), treatment of CCl4-damaged murine renal tissue with propolis led to better kidney structure and less glomerulus swelling compared to the untreated tissue (93). In the renal tissues of diabetic rats, propolis maintained the thickness of the glomerular basement membrane. Untreated diabetic rats showed an increase in the thickness of the glomerular basement membrane (89). Propolis decreases apoptosis of renal cells exposed to CCl4 by down-regulating caspase-9 and up-regulating Bcl-2 gene expression (94). Compared to untreated controls, propolis treatment following methotrexate exposure reduced apoptosis in renal cells and lessened renal morphological degradation (95). 

Renal fibrosis and interstitial inflammation are commonly observed histopathological changes in the 5/6 nephrectomy model (Nx). Treatment with red propolis (RP) exhibited a certain degree of prevention against the progression of renal fibrosis, as evidenced by significantly lower fibrosis levels in Nx+RP rats compared to Nx animals. In the Nx model, the presence of hypertension, interstitial fibrosis, and progressive decline in renal function is observed. The sustained decrease in blood pressure levels observed in Nx+RP animals further supports the notable antihypertensive effect of propolis (96). Also, Brazilian red propolis reduces hypertension and renal injury in the 5/6 nephrectomy model (83).

Propolis signiﬁcantly decreases gentamicin-induced tubular injury, collagen deposition, and apoptosis of renal cells (97). Furthermore, propolis signiﬁcantly decreases gentamicin-induced elevated blood urea nitrogen levels. Propolis alleviates proteinuria, serum creatinine retention, glomerulosclerosis, renal macrophage inﬁltration, and oxidative stress in renal ablated rats (83). Propolis extract also has a protective effect in acute kidney injury. By decreasing oxidative stress and increasing endothelial nitric oxide synthase activity, propolis acts as a protective agent against ischemic–reperfusion acute renal injury. After ischemic–reperfusion, propolis-treated renal tissue had a signiﬁcantly lower tubular necrosis score (98). Propolis also prevents the activation of pro-inﬂammatory signaling pathways such as SMAD in the signaling cascades of the TGF-β family, which are involved in the progression of tubule interstitial ﬁbrosis in advanced CKD in animal models (99). 

Propolis also decreases the expression of many inflammatory genes such as Il1b, Vegfa, Adm, Wnt3a, Akt1, Txn1, Cdkn1b, Herpud1, Noxa1, Car9, Hes1, Hes5, Icam1, Wnt5a, and Mapk1, while up-regulating the expression of other inflammatory genes such as Socs3, Cav1, Dab2, Tnf, Rb1, Wnt6, and Calm1, suggesting the complex immune modulatory effects of propolis. Moreover, propolis reduces the migration of immune cells including neutrophils and macrophages, likely by down-regulating CXCL9 and CXCL10 chemokines (100). According to a randomized, double-blind, placebo-controlled trial in CKD patients by Silveira *et al*. (2019), the consumption of 500 mg/day of propolis extract signiﬁcantly reduced proteinuria and the level of monocyte chemoattractant protein-1 (MCP-1) in urine (55). In hemodialysis patients, propolis extract has shown an inhibitory effect on high-sensitivity C-reactive protein (hs-CRP). Propolis is safe for patients with renal disease and no adverse effects are reported (55, 101). According to a study, propolis treatment reestablished catalase and glutathione production in the renal tissue in a cisplatin nephrotoxicity model (102). It is reported that propolis administration decreased the plasma level of malondialdehyde in streptozotocin-induced diabetic rats (103) (Figure 3).

## Conclusion

Propolis is a rich complex that consists of more than 300 active constituents. Due to its inclusion of a variety of phytochemicals, including CAPE, galangin, chrysin, cardanol, pinocembrin, chicoric acid, as well as phenolic acids, naringenin, and quercetin, propolis exhibits the potential to serve as an efficacious treatment for numerous diseases, by exerting its influence on apoptosis, oxidative stress, and inflammation mechanisms. Consequently, propolis possesses the capability to offer valuable help in the treatment of conditions such as diabetes and cancer. Nonetheless, it is essential to obtain critical knowledge related to its constituents and their interaction with diverse receptors.

## Authors’ Contributions

F S designed the study and wrote the original draft; S SH and R M discussed the results and strategy; A Kh R, H H, and S H supervised the study; S H and R M approved the final version to be published. 

## Funding

This research received no external funding.

## Conflicts of Interest

The authors declare no conflicts of interest.
